# Fractal dimension of antibody‐PEG precipitate: Light microscopy for the reconstruction of 3D precipitate structures

**DOI:** 10.1002/elsc.201900110

**Published:** 2019-11-11

**Authors:** Peter Satzer, Daniel Burgstaller, Walpurga Krepper, Alois Jungbauer

**Affiliations:** ^1^ Department of Biotechnology University of Natural Resources and Life Sciences Vienna Austria

**Keywords:** antibody, engineering, fractal dimension, precipitation, shear stress

## Abstract

Protein and in particular antibody precipitation by PEG is a cost‐effective alternative for the first capture step. The 3D structure of precipitates has a large impact on the process parameters for the recovery and dissolution, but current technologies for determination of precipitate structures are either very time consuming (cryo‐TEM) or only generate an average fractal dimension (light scattering). We developed a light microscopy based reconstruction of 3D structures of individual particles with a resolution of 0.1–0.2 µm and used this method to characterize particle populations generated by batch as well as continuous precipitation in different shear stress environments. The resulting precipitate structures show a broad distribution in terms of fractal dimension. While the average fractal dimension is significantly different for batch and continuous precipitation, the distribution is broad and samples overlap significantly. The precipitate flocs were monofractal from micro‐ to nanoscale showing a random but consistent nature of precipitate formation. We showed that the fractal dimension and 3D reconstruction is a valuable tool for characterization of protein precipitate processes. The current switch from batch to continuous manufacturing has to take the 3D structure and population of different protein precipitates into account in their design, engineering, and scale up.

## 1 INTRODUCTION

Protein precipitation with PEG is used in food and biotechnology for solubility and stability screening 1, 2, 3 and purification of proteins on large scale 4, 5. Only empirical parameters are used for the quality of the precipitation process in scale‐up studies, tech transfer, or changing from batch to continuous operation 6, 7, 8, 9. A common description for precipitate behavior during process development is the Camp number, used as an aging parameter, and related to the density and mechanical strength of the protein precipitate, but this number does not provide information on the actual structure of the precipitate. Fractal dimensions of protein precipitates and agglomerates have been proposed to characterize their structure, but never derived from the 3D structure of the precipitate. PEG precipitation has been described for whey protein recovery 10, 11 and purification of antibodies 6, 9, 11, 12, 13, 14, 15, enzymes, and viruses 16, 17, 18, 19. In almost all applications, the resulting structure of the precipitate itself has been ignored, partly due to the lack of readily available and simple methodology to assess it. Determination of precipitate structures is already used in other industries and typically characterized by the fractal dimension 20, 21, 22, 23, 24, 25, 26. The fractal dimension breaks down complex structures into a single numeral that is easier to work with when different complex structures have to be compared. The concept of fractality has been first introduced in mathematics by Mandelbrot, but nowadays transferred to the engineering disciplines and has been used whenever complex structures have to be characterized, for validating model predictions 27, 28, 29, for following crystallization and precipitation processes 10, 22, 30, and also for diagnostic purposes 31, 32, 33, 34. The measurement of fractal dimension change from application to application, but for protein precipitates typically laser diffraction is used, and the fractal dimension is calculated from the scattering intensity showing fractal dimensions of the resulting structure between two and three 10, 35, 36. Laser diffraction is a valuable tool for the determination, but is limited in the information that can be gained as it yields a single number for the fractal dimension, but does not yield any fractal dimension distribution or complete structure of the precipitate present in the sample. Additionally the method uses dedicated equipment and does not yield a 3D structure of the actual precipitate. Also electron microscopy tomography can be used for 3D structure reconstruction, but is even more time consuming and expensive, but yield excellent resolution and structural information. Due to this lack of easy and accessible methods for structure and fractal dimension determination, the use of this parameter in protein precipitation is still not common. The actual nature of the specific structure is therefore largely neglected by the biotech community at the moment, although the structure of the precipitate itself is very important for process understanding as a whole and even for prediction of the rate limiting processes in protein precipitation and aggregation 10, 37. In addition to the importance of the average structure present in the mixture, the knowledge of the distribution of fractal dimensions in the particle population can substantially improves process understanding. Once the fractal dimension of the population can be determined, this can be correlated to different process parameters, like the shear force during formation of the precipitate or batch or continuous operation or reactions at different scales. Other engineering disciplines, such as the chemical, oil, and mining industry, already use the fractal dimension for controlling the shear force in their processes 10, 22, 25, 28, 37. We developed an easy to use structure determination based on microscopy that is able to render the structure of individual particles to determine a fractal dimension distribution in protein precipitates. These fractal dimensions were then compared to different precipitation conditions to relate the structural information of precipitates to process relevant information.

**PRACTICAL APPLICATION**

The use of microscopy for the determination of fractal dimension populations is an easy and fast method and enhances process understanding and process control. The methodology is easily introduced into laboratories and will offer characterization of complex particle structures for flocculation‐ and precipitation‐based process steps, as well as for process steps where turbidity occurs unexpectedly in the process. It offers a fast, detailed characterization of particle structure populations that is beyond the capacity of current technology such as turbidity measurement, focused beam reflectance measurement, or average fractal dimensions measured by scatter intensity and can rival expensive and time‐consuming methods such as cryo‐TEM tomography. Our study showed the potential of this methodology for enhanced process understanding and that fractal dimension can be established as engineering parameter for the control of precipitation‐based processes for batch and continuous operation.

## 2 MATERIALS AND METHODS

All chemicals were of analytical grade and purchased from Sigma–Aldrich (St. Louis, MO, USA), unless stated otherwise.

### 2.1 Antibody and clarified culture supernatant

The antibody is of IgG2 subtype and was produced by Leck, a Sandoz Company in Chinese hamster ovary (CHO) cell culture. The antibody was provided as cell free supernatant after primary separation as well as in purified form (after protein A purification).

### 2.2 Precipitation (batch, low shear force)

For low shear batch precipitations 6.9 mL of clarified culture supernatant (concentration of 3.2 mg/mL antibody) was mixed with 3.1 mL of 40.0% PEG stock solution and mixed on an end‐over‐end mixer for 20 min with 10 rpm rotational speed. After precipitation, the samples were transferred to the microscope for picture acquisition.

### 2.3 Precipitation (batch, high shear force)

High shear batch precipitations were done in an EasyMax instrument (Mettler Toledo) by mixing 82.2 mL of clarified supernatant (concentration of 3.2 mg/mL antibody) with 37.8 mL of 40.0% PEG while mixing at 500 rpm for 20 min. After precipitation, the samples were transferred to the microscope for picture acquisition.

### 2.4 Precipitation (continuous, low shear force)

Antibody was precipitated in a continuous way by mixing a flow stream of antibody supernatant (5.45 mL/min) in a tubular reactor with a flow stream of 40.0% PEG (2.55 mL/min). The residence time in the tubular reactor was 7.5 min and static mixers were included in the tubular reactor for mixing and to avoid sedimentation. The tubular reactor was built in our laboratory from silicon tubing and helical mixers (Stamixco) with an inner diameter of 4.8 mm. Samples were collected at the end of the reactor and transferred to the microscope for picture acquisition.

### 2.5 Precipitation (continuous, high shear force)

Antibody was precipitated like described above for the low shear force case. After precipitation through the tubular reactor, the precipitate was collected and was subjected to high shear forces on an Äkta flux Tangential Flow Filtration System (GE Healthcare) using a 0.2 µm microfiltration hollow fiber (GE Healthcare) with constant inflow into the reservoir and constant harvest in a continuous operation. The feed flow rate into the system was 15 mL/min, the harvest flow rate was 1.2 mL/min, the permeate flow rate was 13.8 mL, and a recirculation flow rate of 125 mL/min was used using a 50 cm² hollow fiber membrane. The average residence time of the precipitate in this continuous TFF precipitate concentration was 22 min (reservoir volume of 350 mL). The development of this system was published previously by Burgstaller et al. 38.

### 2.6 Microscopy

Generated precipitated samples were transferred to a Leica DMI6000B wide‐field fluorescence microscope using an objective HCX PL APO 100×/1.40 Oil (acquisition parameters: Intensity 1; Exposure 22 ms; Gain 2.5). The *z*‐axis was shifted 0.198 µm after each image to gain a total number of 210 individual images with an *x*/*y* resolution of 0.092 µm per pixel.

### 2.7 Binarization and object detection

Image processing was done using MatLab and the Image Processing Toolbox of MatLab. Each of the collected images was processed for binarization and object detection before all images were combined to reconstruct the 3D structure. For object detection and binarization, an algorithm based on sharp changes in the image, detecting only edges that are in the focal plane of the microscope image was used (MatLab *edge* command with *Prewitt* algorithm). Images were refined by connecting and closing nearby edges. Based on this edge detection the image was binarized and objects at the border of the images were removed. The series of pictures was then combined and object detection was run with standard connectivity (*bwconncomp* command) and the biggest objects were separated into individual files for analysis to exclude small artifact objects in the analysis. Fractal dimensions for each of the objects were calculated using Box Count giving information on the fractal dimension on different scales as well as for the complete structure. Additionally, the surface, total volume, and density were determined for the structures using custom build MatLab scripts.

### 2.8 Cryo‐preparation of PEG6000 precipitated antibody

For cryo‐preparation, the precipitated antibody was transferred into the 100 µm cavity of a 3 mm aluminum specimen carrier. This carrier was sandwiched with a flat 3 mm aluminum carrier and immediately high pressure frozen in an HPF Compact 01 (Engineering Office M. Wohlwend GmbH). The frozen samples were subsequently transferred into a Leica EM AFS‐2 freeze substitution unit (Leica Microsystems). Over a period of 4 days, samples were substituted in a medium of acetone containing 1% Osmium tetroxide. Freeze substitution was performed according to the following protocol: 30–40 h at –90°C; warm up at a rate of 2°C/h to –54°C; 8 h at –54°C; warm up at a rate of 5°C/h to –24°C; 15 h at –24°C; warm up at a rate of 5°C/h to 0°C; and 2 h at 0°C. At 0°C, samples were taken out and washed thrice in anhydrous acetone (on ice) and infiltrated with Agar 100 Epoxy resin (Agar Scientific), in a graded series of acetone and resin over a period of 3 days. Polymerization takes place at 60°C. Ultra‐thin sections with a nominal thickness of 70 nm were cut using a Leica UCT ultramicrotome (Leica Microsystem) and post‐stained with 2% aqueous uranyl acetate and Reynold's lead citrate. Examination regions on the sections were selected at random, examined with an FEI Morgagni 268D (FEI) operated at 80 kV. Digital images were acquired using an 11 megapixel Morada CCD camera (Olympus‐SIS). 

### 2.9 Electron tomography

For room temperature electron tomography, 200 nm sections were made on a Leica UCT ultramicrotome (Leica Microsystems). After collecting the sections on a 50‐mesh Cu/Pd grid (Gilder Grids, Lincolnshire, UK), previously coated with a supporting film of formvar, 10 nm gold (Aurion) was put onto both sides of the section by incubating the grid in a drop of concentrated gold solution for 3 min. Tilt series were acquired at a Tecnai G2 20 microscope (FEI) equipped with an Eagle 4k HS CCD camera (FEI) and operated at 200 kV. Tilt series were collected with a tilting range from –60° to +60° at 1° increment. For data acquisition and processing the IMOD software from the Boulder Laboratory for 3D Electron Microscopy of Cells, University of Colorado Boulder was used.

## 3 RESULTS AND DISCUSSION

### 3.1 Generation of process relevant precipitation samples

We generated process relevant antibody precipitation samples by continuous and batch precipitation using PEG6000. In the past, both continuous and batch purification strategies have been proven technically feasible and economically viable 14, 15, 38. For representative batch precipitation samples, we added a stock solution of PEG6000 to cell free culture supernatant (CFCS) from a fed batch antibody production with an antibody concentration of 3 g/L in a stirred tank reactor to reach 13% PEG. The PEG concentration was already validated to work for a lot of different antibodies in previous work and was therefore used for all experiments without further optimization 14, 15, 38, 39. Two different samples were generated, one using an end‐over‐end mixer to minimize energy input as much as possible (named in this publication as “batch, low shear”) to generate samples with minimal shear force applied during precipitate formation, and one with vigorous mixing (named “batch, high shear”) using a stirred tank. While both processes use different methods for mixing as preparation of minimum shear force samples in a mixed vessel is limited due to settling of the precipitate, the energy input and the shear forces acting on the particles are substantially different and can serve as examples for different processes. These two precipitation samples simulate two different ways of designing a batch precipitation process, generating different precipitate structures according to the relationship established by Camp 40, 41.

In addition to the traditional batch‐based precipitation of proteins, we generated samples in a modern, continuous production approach to establish the influence of modern continuous integrated operation on the structure of protein precipitates. We prepared continuous precipitation samples in a tubular reactor with static mixers without additional shear forces applied to the precipitate (named “continuous, low shear”) and generated additional samples that were subjected to concentration by the use of TFF microfiltration (named “continuous, high shear”) 38. Both samples represent precipitation production scenarios either with or without necessary concentration through TFF‐microfiltration, and therefore, with scenarios relating to either high shear force or low shear force, but in a continuous integrated production scenario. With these four production scenarios implemented, we were able to describe not only the influence of shear force on the structure of the precipitate, but also the influence of switching from batch to continuous mode of production, which will be the future for antibody production. Batch precipitations are already used in industrial production setups, like for instance the Cohn fractionation of blood plasma using ethanol, while continuous precipitations are under development and have been shown to have possible economic benefits depending on the specific use case.

### 3.2 Microscopy and structural reconstruction

**Figure 1:**
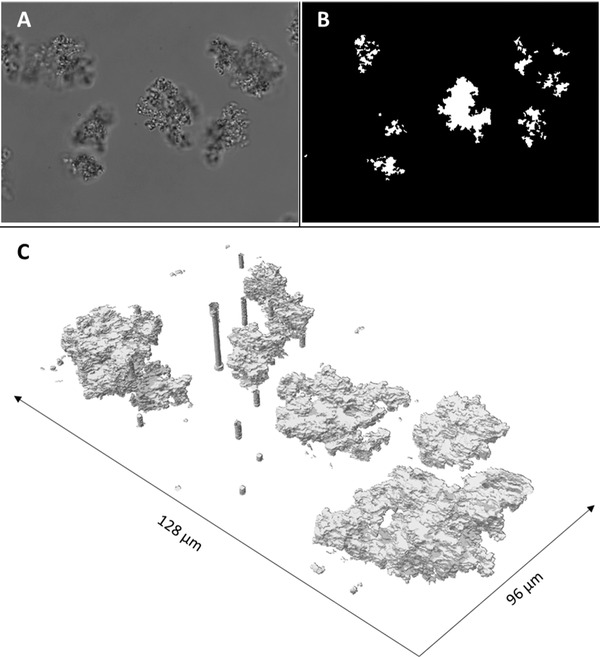
(A) Microscopic image of PEG precipitated antibody particles; (B) binarized image of the precipitate detected by the edge detection algorithm; and image (C) shows the 3D reconstruction of the images series collected

To assess the structure of precipitates beyond the measurement of an overall fractal dimension for the mixture of protein structures it was necessary to reconstruct the 3D structure of individual precipitate particles from microscopy images. To generate the necessary data using a light microscope, an image series was collected of the same precipitate particle, but with a shifting *z*‐axis. Each single image in the series shows the same objects, but with a focal plane at a different height, blurring everything that is not in focus. A representative image of one such series from the batch, low shear production scenario is shown in Figure 1A, clearly showing which parts of the precipitate particle are in focus, and which parts are out of focus. By shifting the focal plane through the particle in the *z*‐direction and using an image processing that makes use of the sharp features in contrast to the blurred features we were able to highlight the outline of the precipitate particle that is in the focal plane and filter out all parts of the particle that are not in focus. We used a readily available algorithm for this from the Matlab image processing toolbox specifically detecting sharp changes in the gray value of the image, highlighting the outline of the precipitate particle. This detected edge was then used to binarize each individual image in the series to contain only the parts of the particle that are in the focal plane (Figure 1B). In contrast to more common and simpler methods for binarization for image processing (like applying a simple threshold to the grayscale image), this method only detects the outline in the focal plane, ignoring all parts of the particle that are not in focus, effectively generating a slice through the particle at the focal plane.

Once all outlines in the image series were generated and the complete image series was binarized to only contain the precipitation particles, the image series was stacked together, spaced from one binarized slice to the next with the distance in the *z*‐axis used for collecting the image series generating a 3D binary array. This binary array is to scale as the total volume recorded is known in all three dimensions (128 µm in length, 96 µm in width, and 50 µm in height) and can therefore be used for a complete 3D reconstruction of the precipitate particles for further analysis (Figure 1C). The generation of such 3D structural reconstructions of precipitates is very fast in comparison to labor intensive cryo‐TEM sample generation as it only requires a microscope and the corresponding MatLab script for image processing of the individual slices and reconstruction of the 3D structure. The image also shows that certain artifacts showing in the reconstruction will be unavoidable, as spots of interference can show up in all images of one series regardless of the *z*‐shift (Figure 1C) resulting in large pillars in the 3D reconstruction that do not correspond to precipitate particles. As these features are very distinct, they can be excluded from the analysis very easily. For further analysis, and in the reconstructions shown in Figure 1, we also excluded very small particles from further analysis to avoid including artifacts due to interference, dust, or similar. We also excluded all particles touching the boundaries of the investigated volume, as they are likely to be truncated. Nevertheless, we were able to include a wide range of particles sizes in our analysis spanning more than three orders of magnitude in regards to the particle volume.

### 3.3 Fractal analysis

We extracted a total of over 150 individual 3D protein particles to be able to use the structures for analyzing the fractality of each individual particle as well as the distribution of fractalities in each sample. For all precipitation particles, the fractal dimension was determined using the Box Count method which uses boxes of different sizes to determine the fractal dimension 42. Using the Box Count method yields a number of boxes containing parts of the precipitate particle which can be plotted in a log plot with the length of the box on the *x*‐axis also called length scale. The length scale is determined by the size of the box used in the box count algorithm and can be best understood by interpreting the fractality being the emerging of new features on different magnifications while looking at the object. The slope can then be used to determine the fractal dimension according to the exponential relationship between counted boxes (*N*), length scale (ε), and fractal dimension (*D*) (equation 1).

(1) $$N\;\propto \varepsilon^{D}$$

The slope of these data points is the fractal dimension, which is 3 for a perfectly filled cube and below 3 for other 3D structures. The resulting slope of the curve can either follow a linear curve, indicating mono‐fractality (an object with one fractal dimension through all length scales) or can follow a nonlinear relationship (referring to an object with multiple different fractalities on different length scales) 43, 44. In addition to the fractal dimension, we determined a particle diameter, particle volume, and density for each precipitate particle individually. The particle diameter was defined as the smallest sphere enclosing the structure of an individual particle. The particle volume was calculated by counting the voxels contained in the particle multiplied by the volume of one voxel. The actual density (mass per volume) of a precipitate particle cannot be calculated from image analysis, we therefore decided to calculate the volume of space filled by precipitate according to image analysis to the volume of the encompassing sphere.

**Figure 2:**
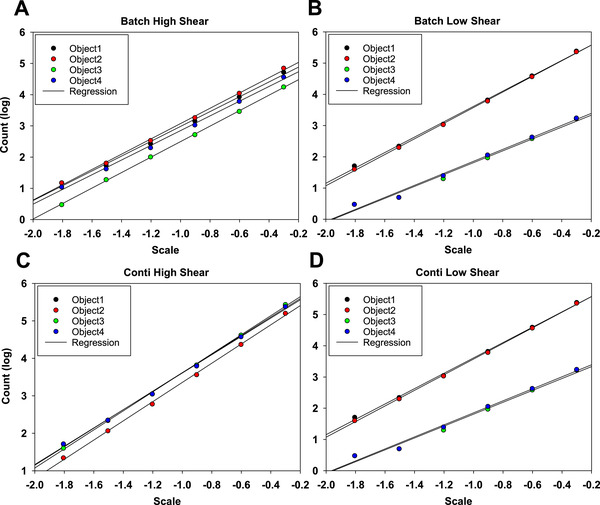
Fractal dimension of different industrial precipitation samples in relation to the scale factor showing monofractality for all samples and conditions, but significant differences between the slope of one sample to the next and also between samples. (Batch High Shear showing precipitate particles generated in batch mode with vigorous mixing, Batch Low shear with minimal mixing. Conti High Shear showing precipitate particles generated in a continuous process concentrated by TFF‐microfiltration, Conti Low Shear without concentration by TFF microfiltration)

We determined the fractality for all objects in relation to different length scales for all four differently prepared samples. Figure 2 shows examples for each of the conditions, showing the log scale counted boxes in relation to the length scale of the boxes used in the Box Count analysis. The analysis yields perfect linear relationships for all precipitate particles, indicating mono‐fractality of all precipitate particles. Features in the protein precipitates are therefore uniform across all length scales accessible by light microscopy. We, therefore, are able to use the slope of the complete curve as accurate measurement of the fractal dimension and do not have to split any analysis in multiple fractalities depending on the length scale under investigation that simplifies further analysis of the data. We can also see that some structures are very similar in slope, but some have quite different fractality, but still being monofractal.

### 3.4 Precipitate structure of individual particles

**Figure 3:**
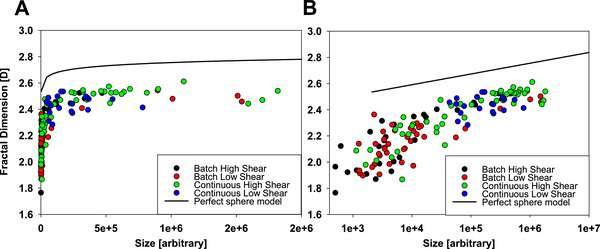
Fractal dimension in relation to the particle size (expressed as number of voxels and demoted as “arbitrary” as it has no unit) for individual PEG precipitate particles for all precipitation conditions tested. Differently prepared precipitations are shown in different colors. The solid line represents a perfect sphere evaluated in the same way as the precipitate particles. Panel (A) shows the relationship in a normal scale for the size of the particle; Panel (B) shows the relationship in a semi‐log scale for the size of the particle

To quantify the influence of switching from a low shear process to a high shear process as well as from a batch process to a continuous process, we plotted the fractal dimension against the particle size expressed as number of voxels of the precipitate particle (Figure 3, panel A in normal plot, panel B in half‐log plot). A clear relationship between particle size and fractality can be observed and we wanted to quantify the influence of the measurement method itself in regards to particle size. A perfect sphere in a perfect analysis would show a fractal dimension of 3, as it is a completely filled 3D perfect structure. In an actual real world analysis, the pixelation of the structure due to the resolution of the microscopy and the box count method itself will generate a surface roughness that is due to the analysis itself and not due to the structure of the object. To quantify this effect, we generated pixelated images of different sizes of perfect spheres and subjected them to the same analysis as the precipitate particles (Figure 3, solid line). We can see that indeed the resolution of the light microscopy in combination with the box count method has a size dependent influence on the analysis that is more pronounced for smaller particles than for larger particles. A large part of the difference in fractal dimension of small and large particles can, therefore, be attributed to the analytical method used, and not to an actual difference in structure between large and small particles. This is of further importance when the structure of small and large particles is compared directly, or when samples of different average particle sizes are compared to each other.

The second important observation in these plots is that there is no clear cut difference between the samples generated by different precipitation methods. Neither do precipitate particles generated by batch precipitation bunch together, nor do low or high shear force particles clump together with distinct voids in between. All samples seem to have overlapping populations of particles that might have a tendency in the data shown (as for batch, low shear, it tends to have lower fractal dimension) but do not represent a strong difference between the samples. There could still be clear differences in the average fractal dimension measured in the samples, but the populations of particles clearly overlap strongly both in size and in fractal dimension.

**Figure 4:**
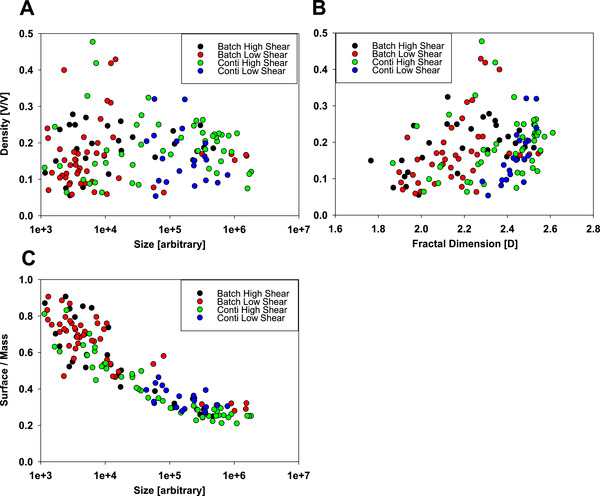
Correlations between particle size, particle density, surface/mass ratio, and fractal dimension for differently prepared PEG precipitates

To further improve the understanding of fractal dimension in terms of different process conditions, we searched for other possible correlations between density, surface to mass ration, size, and fractal dimension. Figure 4 shows these relationships in half‐log plots and we see a clear relationship between size and surface to mass ratio, which is obvious. With smaller particles, the surface to volume ratio gets larger. The density (in volume of precipitate per volume of encompassing sphere) does not clearly correlate with the fractal dimension of the same particle. In terms of difference from one experiment to the next (from one production scenario to the next), we do not see clear cut differences in this analysis either. So the individual variance from one particle to the next is clearly bigger in terms of fractal dimension and other parameters, than the influence of the production conditions. This does not mean that the mean fractal dimension is the same for differently produced material, but it means that the distribution of fractal dimension is clearly very broad in all samples. This also clearly highlights the importance of methods capable to determine not only a mean fractality, but also a fractality distribution, as this overlapping nature would be missed by the measurement of a mean fractality. The important information of fractality distribution will guide to different engineering solutions, than the use of an average fractality, which will give the impression of one uniform fractality for the whole mixture.

### 3.5 Structure variation in particle populations

**Figure 5:**
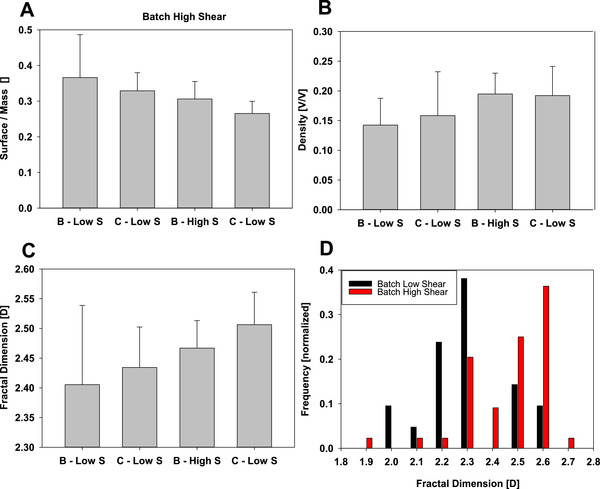
Process scenarios ranked by expected shear stress in relation to different parameters for PEG precipitates: Panel (A) shows the relationship to the surface/mass ratio; Panel (B) shows density (in volume of particle per volume of encompassing sphere); and Panel (C) shows the relationship to the fractal dimension. Panel (D) shows the distribution for two different conditions (batch low and high shear stress) in regards to the fractal dimension for particles larger than 5000 voxels determined by microscopy

To address the question of how much the mean differs from one sample to the next, we used the biggest particles (larger than 5000 voxels) in each data set to minimize the effect of image resolution we showed in Figure 3. Determination of mean fractal dimension and SD is shown in Figure 5 and shows quite substantial deviations from one sample to the next in terms of mean, but also shows that the distribution of fractal dimensions is very large for all investigated parameters and conditions. Ranking the investigated samples in the order of their expected shear forces (batch, low shear; continuous, low shear; batch, high shear; and continuous, high shear), we see a trend of rising average fractal dimension. This is something that can be expected if we assume that loosely bound portions of the precipitate will be sheared off and reorganize until they find a dense enough configuration to withstand the applied shear forces. According to the description by Camp and the formulated Camp Number, this relationship and aging of the precipitates while being subjected to shear forces is both time and shear force dependent 40, 41. According to this description, the Camp number is formulated as shown in Equation (2), where γ is the Camp number, or ageing parameter, *P* is the power input, *V* is the volume of the reactor, ρ is the density of the suspension inside, and *v* is the viscosity. The Camp number, therefore, relates the normalized power input for a reactor of a certain density and viscosity.

(2) $$\gamma ={\left(\frac{P/V}{\rho v}\right)}^{\frac{1}{2}}$$

Therefore, it is expected that this behavior of precipitate aging leading to denser particles will be more pronounced for larger shear forces, leading to a less fractal shape of the precipitate and denser particles. This is also reflected by the decrease of surface area per mass, as looser particles will have more surface area exposed than compact particles. The SDs shown here are not to be confused with SDs from other methods, such as laser diffraction measurements, as in laser diffraction, individual precipitates cannot be resolved and it shows an average of thousands of individual particles. Here in our case, we see the deviation not from one measurement of a thousand particles to the next, but from each individual particle to the next, so higher deviations from the mean have to be expected as they represent the fractality distribution in the sample and not the measurement error.

To investigate the population of particles of different process scenarios, we used the fact that we resolve each particle individually to build up a fractal dimension distribution of the population, something that cannot be done with any other method (Figure 5D). Cryo‐TEM is very tedious to acquire a large number of individual precipitate particles and other methods only determine a mean fractal dimension in a mixture. We compared the population of all precipitate particles larger than 5000 voxels of the conditions of least shear force (batch, low shear) and condition of highest shear force (continuous, high shear) in terms of fractal dimension distribution. While we see a quite big overlap between these two populations, we clearly see a shift of the maximum to higher fractal dimensions for the process using higher shear forces, which is also represented in the higher average fractal dimension. This highlights the importance of determining a fractal dimension distribution in comparison to determining an average fractal dimension.

### 3.6 Rate limiting step for precipitation

The fractal dimension of precipitates can be used to describe the complex structure of precipitates, but it has also been used in the literate to infer the rate limiting step of the precipitation from the resulting fractal dimension. Robinson et al. 37 described the kinds of rate limitation and resulting fractal dimension and according to the literature, one would expect rather low fractal dimensions of 1.7 for a reaction limited precipitation, while a fractal dimension of 2.4 would be expected for diffusion limited precipitation. These results have to be compared to samples with low to no shear force because the analysis done by Robinson et al. does not take shear stress or agitated mixing into account. The lowest shear conditions in our test samples is the batch prepared precipitation with minimal agitation. This sample showed an average fractal dimension of about 2.4, which is in agreement to a diffusion‐limited precipitation. This is in line with theory, as proteins, and especially antibodies are big macromolecules and diffuse very slowly, so it can be expected that the diffusion rate is orders of magnitude lower than the reaction rate upon precipitation.

### 3.7 Comparison of fractality on micro‐ and nanoscale

**Figure 6:**
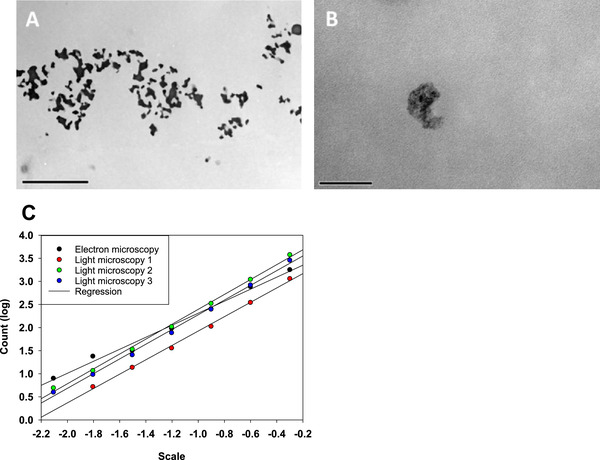
Panel (A) shows a cryo‐TEM slice of the PEG precipitate (scale bar 5 µm); Panel (B) shows a close up of a small precipitate and its internal structure (scale bar 200 nm); Panel (C) shows the comparison of fractal dimension (slope) of TEM image analysis and microscope image analysis

As light microscopy has limited resolution and does not allow imaging of internal structures, we analyzed images obtained from cryo‐TEM tomography. Smaller internal structures not visible by light microscopy could have a different fractal dimension than the larger outer structures. We, therefore, performed high pressure freezing TEM and resin substitution of precipitate samples prepared from batch precipitation with minimal agitation. Nano‐scale structures visualized by cryo‐TEM were compared to structures from microscale with light microscopy. The slices of the tomography were taken in 60 nm distances and the internal structure as well as overall shape of the precipitate was well resolved in TEM (Figure 6). Figure 6A shows the microstructure of the precipitate that agrees very well with the light microscopy images in Figure 1. The outline of the structure as well as the internal structure can be visualized, which is obscured to the light microscopy. Structures that are in the middle of the particle that are obscured by interference in the light microscopy are completely preserved and resolved in the cryo‐TEM. Additionally we can identify internal nano‐structures on closer inspection in the TEM‐images (Figure 6B) that do not show any significant regularities. Dense and less dense regions seem to bunch together in a random arrangement strengthening the idea that precipitation is a random fractal aggregation that does not follow any specific regularity. To deepen the understanding of the actual 3D precipitate structure, we also performed tomography of a 400 nm thick slice (not shown) that confirms that structures seen in the sliced precipitate (Figure 6A) are interconnected in 3D.

In order to compare the fractal dimension on microscale captured by light microscopy with the fractal dimension on nanoscale captured by the cryo‐TEM, we intended to use the structure provided by the tomography. Unfortunately the maximum thickness achievable was 400 nm slices that are insufficient to capture complete particles in the sample. Therefore, we compared the 2D fractality of the pictures collected by TEM with the 2D fractality obtained by one image from the light microscopy (Figure 6 C). We treated the TEM images the same way as the images from light microscopy and evaluated them by using box‐count. In this case, the slope is expected to show a fractal dimension of 2 for a complete solid structure, and fractal dimensions below 2 for other structures. The resulting curves are shown normalized to the length scale of the image to compare light and electron microscopy directly. We can see in the analysis that the slope for both images is very similar, which indicates that the monofractality seen using the 3D structures generated by light microscopy also extends down to the scale captured by TEM. Although the analysis fits the expectation, this finding has to be taken with caution, as we know from the previous analysis that the difference from one particle to the next can be substantial even if they are prepared in the same precipitation mixture and under the same conditions. What can be inferred is that the rate limiting step for all features in the protein precipitate is the diffusion, regardless if it is for the generation of small features (expected to happen at the start of precipitation) or large features (are expected to form later during maturation).

## 4 CONCLUDING REMARKS

We hope that the presented light microscopy based determination of fractal dimensions for individual particles will help laboratories to implement fractal dimension as an engineering parameter into their studies with minimal cost. Reconstruction of complete 3D structures of individual precipitate particles from light microscopy pictures is a simple and fast method for precipitate characterization. The influence of different precipitation conditions such as increasing shear force and precipitate aging can be determined with this method as a distribution of fractalities rather than a median fractality. As a by‐product of this development, we were able to confirm monofractality for all process conditions, be it high shear force or continuous manufacturing. The fast, cheap, easy, and accessible determination of fractal dimension distributions of protein precipitates can be used in any lab and we hope that the method will serve to establish fractal dimension as an engineering parameter for process development in the biotechnology community.

## CONFLICT OF INTEREST

The authors have declared no conflict of interest.
